# Association of Thyroid Function With Restenosis and Long-Term Outcomes After Drug-Coated Balloon Angioplasty in Euthyroid Patients With Coronary Heart Disease

**DOI:** 10.31083/RCM46009

**Published:** 2026-02-25

**Authors:** Yixin Duan, Chenyang Xu, Qian Zhao, Jun Gu, Junfeng Zhang, Yang Zhuo, Huili Zhang

**Affiliations:** ^1^Department of Cardiology, Shanghai Ninth People's Hospital, Shanghai JiaoTong University School of Medicine, 200011 Shanghai, China; ^2^Department of Cardiology, The First People's Hospital of Lancang Lahu County, 665600 Pu'er, Yunnan, China

**Keywords:** coronary disease, balloon angioplasty, coronary restenosis, thyroid hormones

## Abstract

**Background:**

The role of euthyroid hormone levels in clinical outcomes after drug-coated balloon (DCB) angioplasty in patients with coronary heart disease (CHD) remains unclear. Thus, this study aimed to explore the relationship between thyroid function and the risk of restenosis at one year, as well as the prognosis over five years in euthyroid patients with CHD following DCB angioplasty.

**Methods:**

This prospective study evaluated 189 euthyroid CHD patients who underwent successful DCB angioplasty. Coronary angiographic follow-up was performed 9–12 months post-procedure to assess the status of target lesions, with restenosis defined as ≥50% reduction in luminal diameter. All patients underwent five-year clinical follow-ups, during which major adverse cardiovascular events (MACEs) were recorded.

**Results:**

Following angiographic follow-ups, patients were categorized into two groups: those with restenosis (n = 66) and those without (n = 123). At baseline and during the follow-up, the restenosis group demonstrated significantly higher levels of thyroid-stimulating hormone (TSH), lymphocytes, hemoglobin A1c (HbA1c), lipoprotein(a), and platelet count, along with lower free triiodothyronine (FT3) levels. Multivariable logistic regression analysis revealed that the TSH levels both at the baseline (odds ratio (OR) 1.607, 95% confidence interval (CI) 1.238–2.085, *p* < 0.001) and angiographic follow-up (OR 2.970, 95% CI 2.000–4.411, *p* < 0.001) were independently associated with an increased risk of post-DCB restenosis. Furthermore, patients in the high TSH tertile had a 90% increased risk of MACEs during the 5-year follow-up period (hazard ratio (HR) 1.922, 95% CI 1.343–2.750, *p* < 0.001) compared with those in the low TSH tertile.

**Conclusions:**

A high-normal TSH level within the euthyroid range was strongly associated with an increased 1-year restenosis risk and decreased 5-year MACE-free survival following DCB angioplasty in CHD patients.

## 1. Introduction

Drug-coated balloons (DCBs) are innovative coronary devices that combine a semi-compliant balloon catheter with a specialized coating to deliver antiproliferative drugs (e.g., paclitaxel or sirolimus) directly to the vessel wall during percutaneous coronary intervention [1]. DCBs offer an alternative to conventional balloon angioplasty and stenting in specific anatomic settings. Nowadays, this “leave-nothing-behind” approach has gained increasing clinical adoption across various settings. DCBs have been established as the standard treatment for in-stent restenosis (ISR), regardless of stent type [2, 3]. DCBs have also shown promising efficacy and safety in expanding applications, including small vessels, de novo lesions, and patients with high bleeding risk [2, 3]. Ongoing investigations are exploring potential roles in more complex scenarios, including bifurcation lesions, large-vessel disease, diabetic patients, and acute coronary syndrome (ACS) [2, 3]. The therapeutic efficacy of DCBs depends on multiple determinants, including drug coating, excipients, lesion characteristics, preparation technique, and procedural parameters (inflation pressure/temperature) [4]. However, persistent challenges remain, as certain patients experience binary restenosis of target lesions and recurrent cardiovascular events post-DCB treatment [4]. These challenges underscore the need for further investigation into clinical factors that influence outcomes of the DCB angioplasty.

It is well-established that both clinical and subclinical hypothyroidism increase the risk of atherosclerosis and myocardial infarction [5, 6]. Further studies have explored the link between euthyroid function and cardiovascular disease (CVD) risk, demonstrating that low-normal thyroid function was associated with more severe atherosclerosis in the carotid and coronary arteries [7, 8, 9, 10]. Moreover, some studies suggested that high-normal thyroid-stimulating hormone (TSH) levels conferred an elevated risk of coronary heart disease (CHD) and adverse clinical outcomes [11, 12, 13, 14]. Despite these findings, the role of euthyroid hormone levels in CHD patients undergoing DCB angioplasty remains poorly characterized. Given this gap, this study sought to evaluate the association of free triiodothyronine (FT3), free tetraiodothyronine (FT4), and TSH levels with 1-year restenosis risk and 5-year clinical outcomes in euthyroid CHD patients following DCB angioplasty.

## 2. Methods

### 2.1 Participants and Study Design

A prospective observational study was conducted at Shanghai Ninth People’s Hospital, affiliated with Shanghai Jiao Tong University School of Medicine. The study complied with the Declaration of Helsinki and received approval from the hospital ethics review board (Approval No. SH9H-2019-T160-2). All participants signed informed consent before enrollment. In total, 252 consecutive patients with CHD who underwent successful DCB angioplasty for ISR or de novo small vessels (diameter of the target vessel ≤3 mm) were evaluated for enrollment in this study. Coronary angiographic follow-up was performed 9–12 months after the DCB procedure to assess the status of the target lesions. DCB stenosis was defined as ≥50% reduction in luminal diameter post-intervention. Patients with cardiomyopathy, severe heart failure, valvular heart disease, thyroid disease, serious respiratory diseases, severe hepatic and renal dysfunction, malignant tumors, autoimmune diseases, and infectious diseases were excluded from the study.

### 2.2 Data Collection and Follow-up

Demographic data, angiographic findings, and laboratory results were collected at baseline (admission for DCB angioplasty) and during angiographic follow-up (9–12 months after DCB angioplasty). Following the DCB procedures, all patients were followed up at outpatient clinics every three months for five years. For patients unable to attend scheduled clinic appointments, telephone interviews were conducted to ensure follow-up. Major adverse cardiovascular events (MACEs), defined as a composite of cardiac death, myocardial infarction, coronary revascularization, and stroke, were systematically documented throughout the follow-up period.

**Fig. 1.**
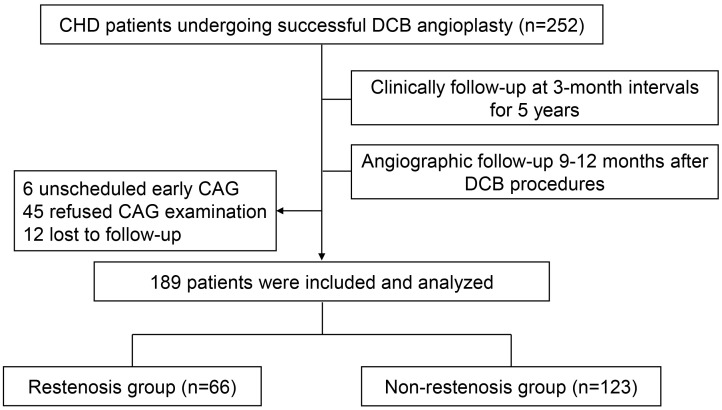
**Schematic workflow of the study**. Abbreviations: CHD, coronary heart disease; DCB, drug-coated balloon; CAG, coronary angiography.

Ultimately, 189 patients who completed both protocol-mandated angiographic follow-up (scheduled for 9–12 months after DCB angioplasty) and the entire five-year clinical follow-up were finally enrolled and analyzed. This group was derived from an initial 252 patients, with exclusions due to unscheduled early angiography (n = 6), refusal of the angiographic follow-up (n = 45), and loss to clinical follow-up (n = 12). The patients were divided into restenosis and non-restenosis groups based on the findings at angiographic follow-up. See Fig. 1 for an illustration of the study design.

### 2.3 DCB Angioplasty

All patients received standardized antiplatelet therapy before DCB angioplasty. All procedures utilized paclitaxel-coated DCBs and were performed by experienced interventional cardiologists in accordance with the International DCB Consensus Group guidelines [2]. Target lesions were routinely predilated by using an optimal-sized balloon with a balloon-to-artery ratio of 0.8–1:1. To achieve optimal lesion preparation, noncompliant balloons, cutting and scoring balloons, or atherectomy devices were used in this study. After proper lesion preparation, DCB was inflated for at least 60 seconds with its nominal pressure, taking care to extend the DCB at least 2 mm beyond the pre-dilation balloon length. Bailout stenting was immediately performed if a flow-limiting dissection or >30% residual stenosis occurred either after lesion preparation or after DCB inflation.

After the DCB procedure, all patients took aspirin and a P2Y12 inhibitor for 1–3 months according to their bleeding risk. While 184 patients completed at least three months of dual antiplatelet therapy, five patients discontinued treatment after one month owing to bleeding complications, including gastric ulcer, erosive gastritis, bladder bleeding, recurrent gingival bleeding, and subconjunctival hemorrhage (one case each). All patients received guideline-directed medical therapy, which included statins, β-blockers, renin-angiotensin system inhibitors, nitrates and other lipid-lowering drugs.

### 2.4 Laboratory Assay 

Following a 12-hour overnight fast, blood samples were obtained for hematological, biochemical, and thyroid function analyses at baseline (pre-operative) and during angiographic follow-up. Complete blood counts were performed using an automated Coulter LH780 Hematology Analyzer (Beckman Coulter, Limerick, Co. Clare, Ireland). Serum lipid profiles were measured using a Siemens Advia 2400 biochemical autoanalyzer (Siemens, Tokyo, Japan). Thyroid function parameters were assessed via direct chemiluminescence immunoassay, with TSH reference ranges established at 0.56–5.91 µIU/mL. Additional biomarkers, including troponin I, C-reactive protein (CRP), and B-type natriuretic peptide (BNP), were analyzed using a fully automated chemiluminescent immunoassay system.

### 2.5 Statistical Analysis

Continuous variables, presented as median [first quartile, third quartile], were compared with the Mann-Whitney U test for between-group differences and the Wilcoxon signed-rank test for within-group differences. Categorical variables, presented as percentages, were compared using the chi-square test. Independent factors associated with DCB restenosis were identified using multivariable logistic regression analysis. The odds ratios (OR) and 95% confidence intervals (CI) were calculated. Clinical and laboratory variables with a *p*-value less than 0.05 in the univariate analysis were identified as candidates and subsequently included in the multivariable logistic regression. A multivariable Cox proportional hazards regression analysis was performed to estimate the hazard ratio (HR) for the 5-year MACE risk. All data were statistically analyzed using SPSS 23.0 software (IBM Corp., Chicago, IL, USA).

## 3. Results

### 3.1 Demographic and Clinical Characteristics

Between April 2017 and June 2020, we screened 252 consecutive patients with CHD who underwent successful DCB angioplasty. Of these, 189 patients completed both the protocol-specified coronary angiographic follow-up (9–12 months after DCB procedures) and the entire five-year clinical follow-up, constituting the final study population. Based on follow-up angiographic findings, patients were categorized into two groups: the restenosis group (n = 66) and the non-restenosis group (n = 123). Baseline demographic and clinical characteristics are presented in Table 1. Patients in the restenosis group were more likely to be a higher proportion of smokers (45.5% versus 29.3%, *p* = 0.020), hypertension (75.8% versus 62.6%, *p* = 0.046), and diabetics (51.5% versus 36.6%, *p* = 0.034). They also had a lower frequency of statin use (86.4% versus 95.1%, *p* = 0.035). No significant difference was noted between the two groups regarding age, sex, body mass index (BMI), prior history of CHD, myocardial infarction, or percutaneous coronary intervention (PCI)/percutaneous transluminal coronary angioplasty (PTCA), indications for DCB, and types of CHD at admission.

**Table 1.** **Baseline characteristics of the study population**.

		Restenosis group (n = 66)	Non-restenosis group (n = 123)	*p* value
Age (years)		67.50 (62.00, 73.25)	69.00 (63.00, 76.00)	0.288
BMI (kg/m^2^)		24.39 (21.59, 26.54)	23.66 (21.03, 25.83)	0.106
Male (%)		57 (86.4)	97 (78.9)	0.242
Current smoker (%)		30 (45.5)	36 (29.3)	0.020
Family history of CVD		32 (48.5)	55 (44.7)	0.366
Medical history				
	CHD (%)	38 (57.6)	66 (53.7)	0.611
	Myocardial infarction (%)	3 (4.5)	14 (11.4)	0.117
	PCI/PTCA (%)	16 (22.7)	43 (35.0)	0.130
	Hypertension (%)	50 (75.8)	77 (62.6)	0.046
	Diabetes mellitus (%)	34 (51.5)	45 (36.6)	0.034
	Ischemic stroke (%)	11 (16.7)	18 (14.6)	0.432
Diagnosis at admission				
	ACS	25 (37.9)	33 (26.8)	0.116
	Stable angina pectoris	41 (62.1)	90 (73.2)	0.137
Indications for DCB				
	ISR (%)	7 (10.6)	22 (17.9)	0.186
	Small vessel lesions (%)	59 (89.4)	101 (82.1)	0.210
Medications				
	Dual antiplatelet therapy (%)	66 (100)	123 (100)	1.000
	Ticagrelor (%)	15 (22.7)	18 (14.6)	0.162
	β-blocker (%)	38 (57.6)	73 (59.3)	0.467
	CCB (%)	5 (7.6)	12 (9.8)	0.417
	ACEI/ARB (%)	19 (28.8)	41 (33.3)	0.319
	Nitrates (%)	9 (13.6)	24 (19.5)	0.209
	Stains (%)	57 (86.4)	117 (95.1)	0.035
	Other lipid lowering drugs (%)	4 (6.1)	11 (8.9)	0.485

Data are presented as percentages or median [first quartile, third quartile] or number (%) of subjects. Abbreviations: ACS, acute coronary syndrome; BMI, body mass index; ACEI, angiotensin converting enzyme inhibitor; ARB, angiotensin II receptor blocker; CCB, calcium channel blocker; ISR, in-stent restenosis; PCI, percutaneous coronary intervention; PTCA, percutaneous transluminal coronary angioplasty; CVD, cardiovascular disease.

### 3.2 Changes in Laboratory Profiles and Thyroid Function 9–12 Months After DCB Angioplasty

Thyroid function and laboratory profiles, including Troponin I, BNP, blood cell counts, lipid levels, and inflammatory markers, were assessed at baseline and during angiographic follow-up (Table 2). At baseline, most parameters were comparable between the two groups. However, the restenosis group exhibited significantly higher levels of HbA1c, lymphocyte proportion, lipoprotein a (Lp(a)), and TSH, along with a lower level of FT3, compared to the non-restenosis group.

**Table 2.** **Changes in hematological and laboratory parameters 9–12 months after DCB angioplasty**.

	Restenosis group (n = 66)			Non-restenosis group (n = 123)			*p* value (Baseline comparison)	*p* value (Follow-up comparison)
	Baseline	Angiographic follow-up	*p* value	Baseline	Angiographic follow-up	*p* value		
HbA1c (%)	6.60 (6.00, 7.70)	6.30 (5.90, 7.70)	0.202	6.20 (5.40, 7.00)	6.20 (5.70, 7.20)	0.330	0.002	0.024
Troponin I (ng/mL)	0.01 (0.01, 0.0625)	0.01 (0.00, 0.01)	<0.001	0.01 (0.00, 0.03)	0.01 (0.00, 0.01)	<0.001	0.077	0.788
BNP (pg/mL)	63.00 (32.00, 232.75)	44.00 (19.00, 114.50)	<0.001	54.00 (22.50, 125.00)	42.00 (22.00, 94.00)	0.005	0.066	0.661
CRP (mg/L)	1.77 (0.97, 4.77)	1.35 (0.61, 2.82)	0.353	1.28 (0.72, 3.06)	1.28 (0.40, 2.72)	0.086	0.081	0.255
Hemoglobin (g/L)	145.00 (130.00, 151.25)	140.00 (130.50, 152.00)	0.057	138.50 (127.00, 148.00)	137.00 (125.75, 152.00)	0.563	0.087	0.660
WBC (×10^9^/L)	6.22 (5.21, 8.55)	5.91 (5.19, 6.90)	0.001	6.62 (5.60, 7.72)	6.70 (5.64, 7.80)	0.726	0.540	0.001
Neutrophil (%)	65.20 (59.93, 74.85)	67.20 (62.83, 72.90)	0.746	64.50 (59.15, 70.10)	66.35 (60.80, 72.30)	0.004	0.141	0.238
Lymphocyte (%)	23.50 (17.10, 30.33)	21.70 (16.88, 26.45)	0.057	25.55 (20.68, 30.73)	24.05 (18.30, 30.13)	0.001	0.048	<0.001
Platelets (×10^9^/L)	183.50 (161.75, 229.25)	182.50 (148.75, 221.25)	0.060	208.00 (182.00, 247.50)	202.50 (172.75, 246.00)	0.019	<0.001	<0.001
TC (mmol/L)	3.715 (3.11, 4.82)	3.20 (2.89, 4.00)	<0.001	3.96 (3.25, 4.92)	3.01 (2.24, 3.90)	<0.001	0.514	0.004
TG (mmol/L)	1.51 (1.09, 1.94)	1.22 (0.84, 1.65)	0.020	1.54 (1.20, 2.25)	1.26 (0.95, 1.85)	<0.001	0.177	0.574
HDL-C (mmol/L)	0.91 (0.77, 1.21)	0.90 (0.75, 1.14)	0.097	0.98 (0.80, 1.16)	1.04 (0.89, 1.23)	<0.001	0.656	<0.001
LDL-C (mmol/L)	2.47 (1.87, 3.22)	1.70 (1.22, 2.09)	<0.001	2.54 (1.88, 3.44)	1.78 (1.16, 2.62)	<0.001	0.655	0.455
Lp(a) (mg/L)	182.50 (108.00, 440.00)	193.00 (114.50, 322.50)	0.757	92.00 (60.00, 222.75)	100.00 (44.75, 276.00)	0.765	<0.001	<0.001
ApoA1 (g/L)	1.07 (0.96, 1.26)	1.13 (0.98, 1.25)	0.553	1.13 (0.99, 1.28)	1.14 (1.03, 1.28)	0.910	0.224	0.427
ApoB (g/L)	0.73 (0.57, 0.92)	0.53 (0.46, 0.70)	<0.001	0.79 (0.63, 0.96)	0.56 (0.40, 0.78)	<0.001	0.317	0.972
ApoA1/B	1.48 (1.26, 1.84)	2.02 (1.37, 2.63)	<0.001	1.42 (1.17, 1.83)	2.17 (1.51, 3.00)	<0.001	0.606	0.072
ApoE (mg/dL)	3.16 (2.69, 4.66)	2.99 (2.39, 3.42)	<0.001	3.64 (2.76, 4.52)	3.03 (2.29, 3.80)	<0.001	0.271	0.579
TSH (µIU/mL)	2.34 (1.77, 3.14)	2.70 (1.94, 3.23)	0.216	1.79 (1.16, 1.83)	1.65 (1.22, 2.25)	0.002	<0.001	<0.001
FT3 (pg/mL)	2.80 (2.51, 3.08)	2.78 (2.52, 3.04)	0.798	2.97 (2.64, 3.26)	2.98 (2.74, 3.26)	0.215	0.002	<0.001
FT4 (ng/dL)	0.89 (0.80, 0.96)	0.86 (0.76, 0.96)	0.197	0.86 (0.78, 0.95)	0.86 (0.77, 0.95)	0.755	0.399	0.948
TT3 (ng/mL)	0.86 (0.73, 0.99)	0.85 (0.74, 0.98)	0.801	0.86 (0.72, 1.01)	0.85 (0.74, 0.99)	0.516	0.734	0.976
TT4 (µg/mL)	8.79 (7.58, 9.77)	9.22 (7.60, 9.96)	0.012	8.75 (7.74, 9.87)	8.91 (7.80, 10.21)	0.084	0.504	0.767

Data are presented as percentages or median [first quartile, third quartile]. Abbreviations: BNP, B-type natriuretic peptides; CRP, C-reactive protein; LDL-C, low density lipoprotein cholesterol; HDL-C, high density lipoprotein cholesterol; Lp(a), lipoprotein a; TC, total cholesterol; TG, triglyceride; TSH, thyroid-stimulating hormone; TT3, total triiodothyronine; FT3, free triiodothyronine; TT4, total tetraiodothyronine; FT4, free tetraiodothyronine; WBC, white blood cell; HbA1c, hemoglobin A1c.

After an average of nine months of standardized treatment, lipid profiles significantly improved in both groups, with marked reductions in triglyceride (TG), total cholesterol (TC), low density lipoprotein cholesterol (LDL-C), ApoB, and ApoE at angiographic follow-up. Additionally, the non-restenosis group showed a significant increase in high density lipoprotein cholesterol (HDL-C). Changes in thyroid function were also observed between baseline and follow-up. In the non-restenosis group, TSH levels decreased significantly, while FT4 levels increased slightly. In contrast, the restenosis group exhibited no significant changes in thyroid hormones or TSH, except for FT4.

Furthermore, at angiographic follow-up, substantial differences were observed between the two groups in terms of HbA1c, inflammatory markers, lipid profiles, and thyroid function. Compared to the non-restenosis group, the restenosis group displayed higher levels of white blood cell (WBC), lymphocyte proportion, platelet count, TC, Lp(a), and TSH, but lower levels of HDL-C and FT3.

### 3.3 TSH and the Restenosis Risk After DCB Angioplasty

To identify potential factors associated with 1-year restenosis following DCB angioplasty, we conducted a univariate logistic regression analysis. As presented in Table 3, several factors were significantly associated with restenosis after DCB angioplasty. These included clinical characteristics such as smoking, diabetes mellitus, hypertension, as well as statin use. Additionally, hematological indices, including WBC, lymphocyte percentage, platelet count, lipid profiles (TC, HDL-C, Lp(a)), and thyroid function (measured by TSH, FT3 levels) at baseline or during angiographic follow-up, were also identified as important factors.

**Table 3.** **Risk factors for 1-year restenosis after DCB angioplasty**.

		Univariable logistic regression			Multivariable logistic regression		
		OR	95% CI	*p* value	OR	95% CI	*p* value
Model 1 (baseline)							
	Diabetes mellitus	1.699	1.051–2.748	0.031	0.994	0.525–1.881	0.986
	Hypertension	1.641	0.963–2.797	0.069	1.752	0.945–3.248	0.075
	Current smoker	2.014	1.154–3.515	0.014	3.281	1.672–6.438	0.001
	Statins	0.415	0.176–0.975	0.044	0.336	0.114–0.990	0.048
	HbA1c (%)	1.239	1.050–1.462	0.011	1.085	0.875–1.347	0.457
	Lymphocyte (%)	0.967	0.939–0.995	0.023	0.983	0.950–1.018	0.341
	Platelet (×10^9^)	0.991	0.986–0.996	<0.001	0.988	0.981–0.994	<0.001
	Lp(a) (mg/L)	0.254	0.158–0.406	<0.001	1.001	1.000–1.003	0.019
	TSH (µIU/mL)	1.658	1.341–2.049	<0.001	1.607	1.238–2.085	<0.001
	FT3 (pg/mL)	0.414	0.243–0.707	0.001	0.570	0.301–1.078	0.024
Model 2 (angiographic follow-up)							
	Diabetes mellitus	1.699	1.051–2.748	0.031	0.783	0.373–1.646	0.519
	Hypertension	1.641	0.963–2.797	0.069	1.055	0.522–2.130	0.882
	Current smoker	2.014	1.154–3.515	0.014	3.450	1.614–7.269	0.001
	Statins	0.415	0.176–0.975	0.044	0.131	0.037–0.458	0.001
	HbA1c (%)	1.227	1.030–1.462	0.022	1.323	1.000–1.749	0.050
	WBC (×10^9^)	0.767	0.653–0.900	0.001	0.664	0.529–0.835	<0.001
	Lymphocyte (%)	0.966	0.935–0.997	0.034	0.947	0.904–0.991	0.019
	Platelet (×10^9^)	0.992	0.987–0.996	0.001	0.996	0.990–1.002	0.166
	TC (mmol/L)	1.420	1.148–1.756	0.001	1.246	0.925–1.679	0.148
	HDL-C (mmol/L)	0.209	0.082–0.532	0.001	0.183	0.055–0.615	0.006
	Lp(a) (mg/L)	1.002	1.001–1.004	0.001	1.002	1.000–1.004	0.086
	TSH (µIU/mL)	3.105	2.232–4.319	<0.001	2.970	2.000–4.411	<0.001
	FT3 (pg/mL)	0.254	0.134–0.480	<0.001	0.660	0.298–1.465	0.307

OR, odds ratio; CI, confidence interval.

Following this, we performed a multivariable logistic regression analysis to evaluate independent factors more comprehensively. After adjusting for confounding variables, TSH levels at both baseline (OR 1.607, 95% CI 1.238–2.085; *p* < 0.001) and follow-up (OR 2.970, 95% CI 2.000–4.411; *p* < 0.001) emerged as independent risk factors for post-DCB restenosis in euthyroid patients with CHD. Other conventional predictors such as diabetes and statin use were also found to be significant. Moreover, platelet count, Lp(a), and FT3 levels at baseline, as well as WBC, lymphocyte proportion, and HDL-C at angiographic follow-up, were also significantly associated with 1-year DCB restenosis risk.

### 3.4 TSH and Long-Term Clinical Outcomes After DCB Angioplasty

**Fig. 2.**
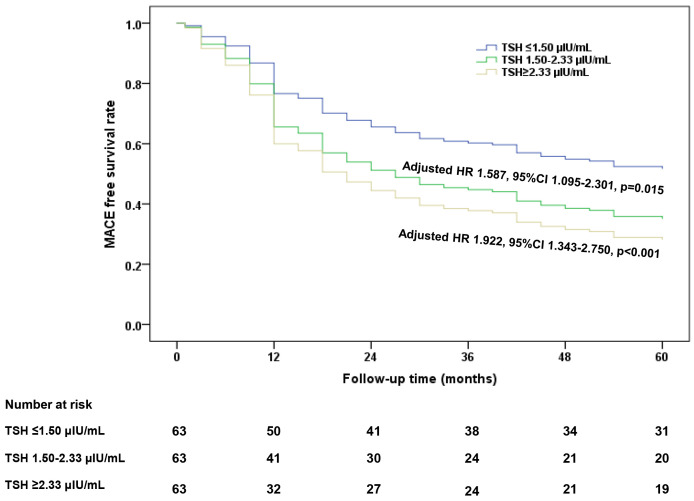
**Cox regression analysis of 5-year MACE-free survival stratified by TSH tertiles in CHD patients undergoing DCB angioplasty**. MACE, major adverse cardiovascular events.

To evaluate the prognostic value of TSH in euthyroid patients undergoing DCB angioplasty, we stratified participants into tertiles based on their baseline TSH levels and followed them for five years. The incidence of MACE showed a progressive decline across TSH tertiles: 69.8% (44/63) in the high tertile (TSH ≥2.33 µIU/mL), 68.3% (43/63) in the intermediate tertile (TSH 1.50–2.33 µIU/mL), and 50.8% (32/63) in the low tertile (TSH ≤1.50 µIU/mL). After adjusting for smoking status in a multivariable Cox regression analysis, patients in the high (adjusted HR 1.922, 95% CI 1.343–2.750, *p* < 0.001) and intermediate (adjusted HR 1.587, 95% CI 1.095–2.301, *p* = 0.015) TSH tertiles exhibited a significantly higher risk of 5-year MACE compared with those in the low tertile (Fig. 2). These findings indicated a 90% elevation in MACE risk for CHD patients with TSH ≥2.33 µIU/mL following DCB angioplasty, compared to those with TSH levels ≤1.50 µIU/mL.

## 4. Discussion

In this small-scale prospective study of euthyroid patients with CHD undergoing DCB angioplasty, serum FT3 and TSH levels, despite being in the normal range, were significantly associated with DCB restenosis along with conventional risk factors such as smoking, statin use, lymphocyte proportion, platelet count, and HDL-C. Notably, patients with baseline TSH levels in the high-normal range had significantly worse 5-year MACE-free survival after DCB treatment compared to those with low-normal TSH levels. These results indicate that variations in thyroid hormone levels, despite falling within the normal reference range, may influence both short-term clinical outcomes (1-year restenosis) and long-term prognosis in CHD patients treated with DCB.

Both clinical and subclinical hypothyroidism are well-recognized risk factors for the development of atherosclerosis [5, 6]. Levothyroxine replacement therapy has been shown to reduce carotid intima-media thickness (IMT) and mitigate cardiovascular risks in patients with subclinical hypothyroidism [15, 16]. However, the association between euthyroid function and atherosclerosis is still debated. Several cross-sectional studies have shown that carotid IMT is independently associated with low FT4 or high TSH levels, even within the normal reference range [7, 8, 9]. Among CHD patients, FT3 levels exhibit an inverse correlation with the severity of coronary artery stenosis, as estimated by coronary angiography (CAG), whereas TSH levels demonstrate a positive correlation [10, 12, 17]. Conversely, in a large cohort of healthy, young, and middle-aged, euthyroid individuals, FT4 and TSH within the low-normal range were demonstrated to be associated with a greater prevalence of subclinical coronary artery disease and more severe coronary artery calcification [18]. Furthermore, research by Chiche *et al*. [19] reported that in patients with dyslipidemia, whether hypothyroidism or euthyroid, neither TSH nor FT4 levels served as independent risk factors for carotid atherosclerosis. They suggested that hypothyroidism did not increase the risk of carotid atherosclerosis when accounting for cardiovascular risk factors such as hypercholesterolemia or hypertension [19]. These conflicting results may stem from variations in study populations or the absence of longitudinal monitoring of thyroid function during follow-up.

Recent studies have explored the relationship between thyroid function and clinical outcomes following PCI. Evidence suggests that subclinical hyperthyroidism does not elevate the risk of adverse cardiovascular events in CHD patients undergoing PCI [20]. Conversely, subclinical hypothyroidism has been correlated with more severe coronary artery lesions and a higher incidence of MACE in non-ST-segment elevation acute coronary syndrome (NSTE-ACS) patients treated with PCI [21]. Furthermore, elevated TSH levels–even within the reference range–have been identified as an independent predictor of higher all-cause mortality in patients undergoing PCI for acute myocardial infarction (AMI) [22]. However, the clinical implications of thyroid hormone variations within the reference range after DCB angioplasty remain poorly understood. Thus far, only Zhang *et al*. [23] have provided preliminary evidence suggesting that low FT3 levels may predict MACE in euthyroid CHD patients after DCB treatment. However, this study has limitations, including its retrospective design and lack of angiographic validation.

In our study, thyroid function was assessed at baseline during angiographic follow-up. At baseline, the restenosis group exhibited significantly higher TSH and lower FT3 levels compared to the non-restenosis group. At the angiographic follow-up after DCB treatment, TSH levels remained unaltered in the restenosis group while showing a decline in the non-restenosis group. Thyroid hormone levels (FT3 and FT4) remained stable in both groups throughout the follow-up period. Consequently, the restenosis group maintained relatively higher TSH and lower FT3 levels than the non-restenosis group. Elevated TSH levels demonstrated significant associations with both 1-year DCB restenosis and 5-year MACE risks, while reduced FT3 levels were independently correlated only with 1-year DCB restenosis risk. After adjusting for smoking status, patients in the high TSH tertile had a 90% higher risk of 5-year MACE compared with those in the low tertile. These findings imply that even within the euthyroid range, variations in TSH levels may significantly influence clinical outcomes in CHD patients following DCB angioplasty. Separately, our cohort was characterized by relatively low levels of Lp(a) and ApoB, reflecting extensive statin use and effective lipid management at baseline. Collectively, these results underscore the clinical relevance of TSH in this contemporary, well-managed patient population.

Multiple mechanisms may explain the association between high-normal TSH levels and increased risks of restenosis and poor long-term outcomes following DCB angioplasty. Population-based studies, both prospective and cross-sectional, have consistently reported that even within the normal range, variations in TSH levels can have significant clinical implications [24, 25]. Furthermore, in euthyroid individuals, elevated TSH has been associated with several cardiovascular risk factors, including hypertension [25, 26], endothelial dysfunction [27], and insulin resistance [28]. These conditions can lead to atherosclerosis and increase the risks of myocardial infarction, stroke, and heart failure [29, 30]. It is important to note that serum TSH levels primarily reflect thyroid-pituitary feedback rather than direct effects of thyroid hormone action in peripheral tissues. Adverse cardiovascular effects may occur even when TSH remains within the conventional reference range. Thus, it calls for re-evaluating TSH reference values in specific clinical conditions, particularly in CHD patients experiencing AMI or undergoing interventional procedures.

As the biologically active form of thyroid hormone, FT3 directly mediates end-organ effects [30]. Low-normal FT3 is associated with increased TC and LDL-C, but also slightly higher levels of HDL-C and lower levels of triglycerides and insulin resistance [30]. Our study found that both baseline and follow-up FT3 levels were correlated with the risk of restenosis following DCB; however, they did not show a significant association with 5-year MACE. These findings highlight the need for large-scale prospective studies to further elucidate the prognostic value of FT3 in predicting long-term outcomes following DCB angioplasty.

Although many studies have established connections between euthyroid dysfunction and cardiovascular risk, it remains uncertain whether thyroid hormone replacement therapy benefits patients with CHD. Current evidence suggests that while thyroid hormone therapy may improve cardiac function, it has not demonstrated significant improvements in long-term clinical outcomes for patients with AMI or those undergoing coronary artery bypass grafting [31, 32]. Additional studies are needed to determine the potential role of thyroid hormone supplementation in CHD management.

The clinical outcomes of DCB angioplasty are influenced by both the antiproliferative agent and the excipient matrix, which affect drug transfer efficiency. The two main agents used in DCBs, Paclitaxel and sirolimus, have distinct pharmacological properties [33]. Paclitaxel’s high lipophilicity enables rapid uptake by the arterial wall and sustained pharmacological activity through microtubule stabilization; however, it can also delay endothelial healing and promote inflammation [33]. In contrast, sirolimus demonstrates favorable healing properties and acts via mammalian (mechanistic) target of rapamycin (mTOR) inhibition. However, its lower lipophilicity and shorter half-life require more advanced delivery technologies [33]. Although preclinical evidence has suggested better endothelial healing with sirolimus-coated balloons, some clinical data demonstrated non-inferior outcomes compared to paclitaxel-coated devices. Researchers are currently conducting large-scale randomized controlled trials to compare these two types of DCBs in a head-to-head manner. Since our study only included paclitaxel-coated balloons, the observed association between TSH and DCB outcomes cannot be generalized to sirolimus-coated balloons.

## 5. Limitations

Additionally, several limitations of our study should be considered. First, as the study population was restricted to CHD patients who underwent DCB angioplasty and completed angiographic follow-up, it limited the generalizability of findings to broader DCB-treated populations. Second, the relatively small sample size and single-center design reduced the statistical power, hindering our ability to detect significant associations and adequately adjust for potential confounders. Third, the absence of reverse T3 and thyroid antibody measurements prevented us from evaluating potential influences from nonthyroidal illness or autoimmune thyroid diseases on the observed inverse relationship between TSH levels and DCB angioplasty outcomes. These limitations highlight the need for caution when interpreting our results and suggest directions for further investigation.

## 6. Conclusions

This study demonstrated that thyroid function, even within the established euthyroid range, is significantly associated with clinical outcomes in CHD patients undergoing DCB angioplasty. Specifically, high-normal TSH levels and low-normal FT3 levels were identified as strong predictors for a higher incidence of restenosis following the procedure. Notably, patients with high-normal TSH concentrations experienced worse 5-year MACE-free survival compared to those with lower TSH levels. These findings indicate that variations in thyroid function, even within the conventional normal range, may impact clinical outcomes after DCB angioplasty. Therefore, we suggest that TSH monitoring could be considered a valuable component of the long-term risk management strategy for this population, pending confirmation in larger studies.

## Availability of Data and Materials

The datasets used and analyzed during the current study are available from the corresponding author on reasonable request.
